# Supplementary material for: Causal Relationship between Aging and Anorexia Nervosa: A White-Matter-Microstructure-Mediated Mendelian Randomization Analysis
Source: Biomedicines. 2024 Aug 16;12(8):1874. doi: 10.3390/biomedicines12081874 (PMC11351342; doi:10.3390/biomedicines12081874)
Supplement: Supplementary file 1 [file biomedicines-12-01874-s001.zip › Supplementary Figure/Figure S4/Singe SNP plot of MR analysis for TL to ubm-b-1756.pdf]

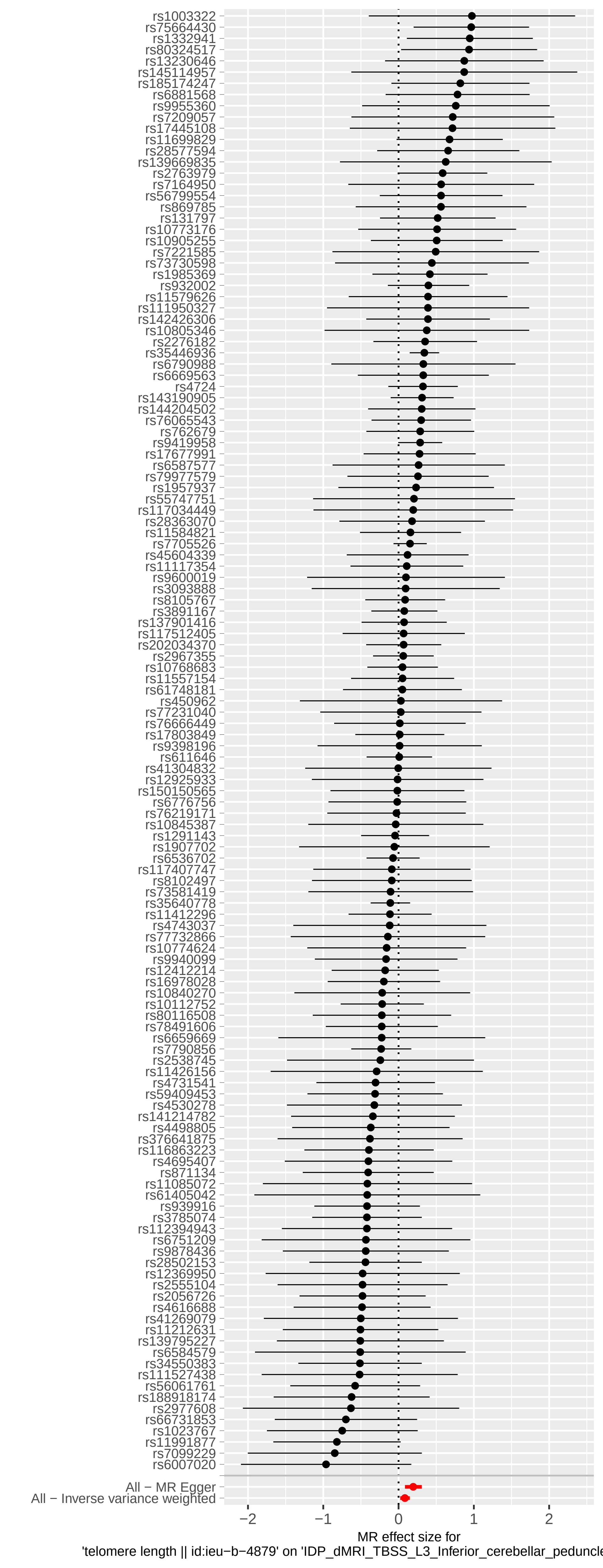
rs1003322
rs75664430
rs1332941
rs80324517
rs13230646
rs145114957
rs185174247
rs6881568
rs9955360
rs7209057
rs17445108
rs11699829
rs28577594
rs139669835
rs2763979
rs7164950
rs56799554
rs869785
rs131797
rs10773176
rs10905255
rs7221585
rs73730598
rs1985369
rs932002
rs11579626
rs111950327
rs142426306
rs10805346
rs2276182
rs35446936
rs6790988
rs6669563
rs4724
rs143190905
rs144204502
rs76065543
rs762679
rs9419958
rs17677991
rs6587577
rs79977579
rs1957937
rs55747751
rs117034449
rs28363070
rs11584821
rs7705526
rs45604339
rs11117354
rs9600019
rs3093888
rs8105767
rs3891167
rs137901416
rs117512405
rs202034370
rs2967355
rs10768683
rs11557154
rs61748181
rs450962
rs77231040
rs76666449
rs17803849
rs9398196
rs611646
rs41304832
rs12925933
rs150150565
rs6776756
rs76219171
rs10845387
rs1291143
rs1907702
rs6536702
rs117407747
rs8102497
rs73581419
rs35640778
rs11412296
rs4743037
rs77732866
rs10774624
rs9940099
rs12412214
rs16978028
rs10840270
rs10112752
rs80116508
rs78491606
rs6659669
rs7790856
rs2538745
rs11426156
rs4731541
rs59409453
rs4530278
rs141214782
rs4498805
rs376641875
rs116863223
rs4695407
rs871134
rs11085072
rs61405042
rs939916
rs3785074
rs112394943
rs6751209
rs9878436
rs28502153
rs12369950
rs2555104
rs2056726
rs4616688
rs41269079
rs11212631
rs139795227
rs6584579
rs34550383
rs111527438
rs56061761
rs188918174
rs2977608
rs66731853
rs1023767
rs11991877
rs7099229
rs6007020
All − MR Egger
All − Inverse variance weighted
−2
−1
0
1
2
MR effect size for
'telomere length || id:ieu−b−4879' on 'IDP_dMRI_TBSS_L3_Inferior_cerebellar_peduncle
